# Epigarcinol and isogarcinol isolated from the root of *Garcinia ovalifolia* induce apoptosis of human promyelocytic leukemia (HL-60 cells)

**DOI:** 10.1186/s13104-015-1596-8

**Published:** 2015-11-23

**Authors:** Constant Anatole Pieme, Pathaleon Ambassa, Emmanuel Yankep, Ajit Kumar Saxena

**Affiliations:** Department of Physiological Sciences and Biochemistry, Faculty of Medicine and Biomedical Sciences, University of Yaoundé I, PO Box 1364, Yaoundé, Cameroon; Cancer Pharmacology Division, Indian Institute of Integrative Medicine, 180001 Canal Road, Jammu, India; Department of Chemistry, Faculty of Sciences, University of Yaoundé I, PO Box 812, Yaounde, Cameroon

**Keywords:** Apoptosis, Cytotoxicity, 7-epigarcinol, Isogarcinol, Antiproliferative, Garcinia ovalifolia

## Abstract

**Background:**

Plants from garcinia genus have been used for centuries against several diseases.

**Objective:**

This study aimed to investigate the mechanism of apoptosis induced by epigarcinol and isogarcinol isolated from the root of *Garcinia ovalifolia* (Clusiaceae) on human promyelocytic leukemia (HL-60 cells).

**Methods:**

Epigarcinol and isogarcinol were isolated from the root of *G. ovalifolia* by using column chromatography method. The antiproliferative property of these molecules and fractions were assessed with 3-(4, 5-dimethylthiazol-2-yl)-2, 5-diphenyltetrazolium bromide (MTT) assay. The light fluorescence microscope was utilized to observe the morphological changes of HL-60 cells after 24 h treatment. Early apoptosis and cell cycle distribution were analyzed by using flow cytometry (FCM).

**Results:**

The results showed that epigarcinol and isogarcinol inhibited the proliferation of HL-60 and PC-3 cells in a concentration-dependent manner with IC_50_ varying between 4 and 76 µg/mL depending on the cell line and the molecule. The apoptosis rate and the number of apoptotic cells significantly increased with the augmentation of the concentration of the molecules. The results of flow cytometry (FCM) indicated that epigarcinol and isogarcinol induced significant G_2_/S arrest of HL-60 cells, the disruption of mitochondrial membrane potential and reactive oxygen species (ROS) generation.

**Conclusion:**

These results indicated that epigarcinol and isogarcinol demonstrated in vitro antiproliferative properties and induce apoptosis of HL-60 cells which is related to the G_2_/S arrest, and it exerts its apoptotic effect through the loosing of mitochondrial membrane potential.

## Background

The global burden of cancer continues to increase largely because of the aging and growth of the world population alongside an increasing adoption of cancer-causing behaviors in economically developing countries. Based on the GLOBOCAN 2012 estimations, about 14.1 million cancer cases and 8.2 million cancer deaths are recorded in 2012. More than half of all cancers (56.8 %) and cancer deaths (64.9 %) in 2012 occurred in less developed regions of the world, and these proportions will increase further by 2025 [1]. Given the fact that the incidence rate of cancer disease is continuously increasing in Africa, a number of effective prevention measures have been introduced in order to substantially reduce the incidence and the mortality. Of all the efforts that have been made, the search for new anticancer compounds in foods or plant medicines is a realistic and promising approach to the prevention and the treatment of cancer [2]. Natural products, including plants, microorganisms and marines, have been considered as valuable sources for anticancer drug discovery [3].With the high speed advances in biomedical research, there has been a growing interest in screening natural products for their potential use in disease prevention and treatment. These natural substances are of interest, as they are potential sources of anticancer compounds with minimal debilitating toxicity and side effects [4]. Because of the different components, herbs and plants may have synergistic activities or buffering toxic effects, mixtures or extracts of herbs might have more therapeutic or preventive activity than alone [5, 6]. Several chemopreventive properties have long been attributed to polyphenolic compounds present in the human diet. One remarkable compound in this list is resveratrol, which possesses a wide range of pharmacological properties, including antiinflammatory, antioxidant, and antiplatelet effects and has been proposed as a cancer chemotherapeutic agent [7, 8].

*Garcinia ovalifolia (G. ovalifolia)* which belongs to Clusiaceae family is growing in lowland forests tropical of Africa, Asia, America and Australia [9]. *G. ovalifolia* is a tree of 10–15 m high, with yellow sticky latex, generally distributed in fringing forests and riverbanks in West and central Africa [10]. The genus Garcinia includes some 200 species found in the tropics, especially Asia and Africa. Plants from Garcinia genus demonstrated several pharmacological proprieties including anti-HIV [10, 11], antioxidant, antibacterial [12], cytotoxic [11, 12], anticancer and antimalarial [13]. The literature review shows that *G. ovalifolia* contains several molecules among which, isoxanthochimol [12, 14], endodesmiadol, canophyllol, canophyllal [12, 15], gallic acid, garcinane [16], 3-methylcheffouxanthone, two new friedelane triterpene derivatives ovalifolone A and B [12]. Epi-garcinol, iso-garcinol and manniflavanone isolated from our plant (*G. ovalifolia)* have also been discovered in other plants extract such as *Garcinia preussii* [17] *Hypericum lanceolatum* [18] *Moronobea coccinea* [19] *Symphonia globulifera* [20] and *Garcinia bancana* [21]. Several biological properties of epi-garcinol, iso-garcinol and manniflavanone have been investigated which included antiplasmodial [20–23], antibacterial [17, 18] and immunosuppressant effects [24].Several biological properties of these molecules have been investigated such as antibacterial, cytotoxicity activity on *Artemia salina* [13] and anti-HIV [25]. However, *G. ovalifolia* has not been studied for its anticancer effects. Therefore, we attempted to investigate the growth-inhibitory and apoptotic effects of *G. ovalifolia extract* and fractions against human cancer cells (HL-60 cells and PC-3).

## Methods

### Collection of plant material

The Stem bark of *Garcinia ovalifolia* (Clusiaceae), was collected in Makenene (Centre region of Cameroon) in December 2010 and identified by Victor NANA of the National Herbarium Cameroon and a sample specimen is deposited on the voucher no. 20854/SRFCam.

### Extraction and isolation of compounds

Air-dried and powdered stem bark of *G. ovalifolia* (2.5 kg) were macerated in methanol (5 L) for 48 h at room temperature. The solution obtained was then filtered through Whatman No. 1 filter paper. The filtrate solution was concentrated under vacuum into a paste to give a dark brown crude extract (150 g). The slurry was made of crude extract (100 g) by dissolving in MeOH, adsorbed on 120 g of silica gel (60–120 mesh) which was subjected to Vacuum Liquid Chromatography (VLC) column packed with 800 g of silica gel (120–200 mesh). Elution was carried out using using hexane/ethyl acetate and ethyl acetate/methanol gradients as eluents at a flow rate of 2 mL/min. Fractions (250 mL each) were collected as follows: pure hexane (fractions 1–5), hexane/ethyl acetate 75/25 (fractions 6–12), hexane/ethyl acetate 50/50 (fractions 13–25),ethyl acetate (fractions 26–33), acetate/methanol 90/10 (fractions 116–125) and methanol (fractions 126–132). These fractions were pooled on the basis of the thin layer chromatography analysis on seven sub-fractions from A to G respectively.

Further chemical investigation of sub- fractions B, C and D was carried out using column chromatography, preparative tin layer chromatography and recrystallization in different solvent yielded three compounds: 7-epigarcinol (250 mg); isogarcinol (25 mg) manniflavanone (40 mg) respectively. All these structures were obtained by the means of spectroscopic analysis including 1D and 2D NMR and mass spectra.

### Cell culture

Human promyelocytic leukemia (HL-60 cells) and prostate cancer (PC-3 cells) were obtained from European Collection of Cells Culture (ECCC), Sigma–Aldrich, India. They were grown in RPMI-1640 medium containing 10 % Fetal bovine serum (FBS),penicillin (100 IU/mL) and streptomycin (100 µg/mL medium).The cells suspension was kept in the incubator (Thermocom Electron Corporation, USA) at 37 °C, 5 % CO_2_; 98 % humidity. Cells were used for different assays during logarithmic growth phase while the untreated control cultures received only the vehicle (DMSO < 0.1 %).

### Cells viability and treatments

The human promyelocytic leukemia (HL-60 cells) and prostate cancer (PC-3 cells) were seeded in 96 different well plates containing 15 × 103 and 6 × 103 cells/100 µL/well, respectively. The cultured cells were then treated (triplicate wells per condition) by adding 100 µL of serial dilutions of the three molecules (7-epigarcinol, isogarcinol and manniflavanone) in DMSO to give a final concentration of 100, 30, 10 and 1 µg/mL. The HL-60 treated cells were incubated immediately while for PC-3 cells, the molecules were added after 24 h of incubation. In addition, the DMSO alone was added to another set of cells as the solvent control (DMSO < 0.1 %). The cells were then incubated for another 48 h prior to the addition of 20 µL of 2.5 mg/mL solution of 3-(4, 5-dimethylthiazol-2-yl)-2, 5-diphenyltetrazolium bromide (MTT) into each well. The incubation was continued for 3 h before the media was removed. A mixture of DMSO (150 µL) was added to each well and mixed to ensure dissolving of the crystal formazan before the absorbance at 570 nm was measured. Three replications of each experiment were performed and fifty percent of inhibitory concentration (IC_50_) of each extract was calculated.

### DNA content and cell cycle phase distribution

HL-60 cells (1 × 10^6^ cells/2 mL/well) were treated with molecules at 20, 50, 100 µg/mL for 24 h. They were harvested and washed with 1 mL of PBS, then centrifuged at 400 g for 5 min at 4 °C. The pellet was suspended in 100 µL of PBS and 900 µL of hypertonic citrate buffer (PI-25 µg/mL, RNAase- 40 µg/mL, sodium citrate-0.1 % and Triton-100X-0.03 %) and incubated at 37 °C in dark for 20 min. Finally, cells were analyzed immediately on flow cytometer FACS Calibur (Becton–Dickinson, USA). The data were collected in list mode on 10,000 events and illustrated in a histogram, where the number of cells (counts) is plotted against the relative fluorescence intensity of PI (FL-2; λem: 585 nm; red fluorescence). The resulting DNA distributions were analyzed by Modfit (Verity Software House Inc., Topsham, ME) for the proportions of cells in G_0_–G_1_, S- phase, and G_2_-M phases of the cell cycle [26].

### Hoechst 33258 staining of cells for nuclear morphology

HL-60 cells (2 × 10^6^ cells/3 mL/well) were treated with isogarcinol and 7-epigarcinol at different concentrations of extract for 24 h. They were collected, centrifuged at 400 g and washed once with PBS. A solution of Hoechst (Hoechst, 10 µg/mL; citric 10 mM; Na_2_HPO_4_ 0.45 M; Tween-20 0.05 %) was added in each tube and kept in the dark at room temperature for 30 min. The mixture was washed with PBS and the pellet suspended in 100 µL of PBS/glycerol (1:1). The solution (10 µL) was poured into the slide and nuclear morphology alterations observed under fluorescence microscope (Olympus ×70, magnification ×20) [26].

### Mitochondrial membrane potential (MMP) assay

HL-60 cells (1 × 10^6^ cells/2 mL/well) were treated with isogarcinol and 7-epigarcinol at different concentrations for 24 h. Thirty minutes before the end of the experiment, the cell culture was treated with Rhodamine-123 (200 nM) and kept in the dark for 30 mn. Cells were collected, centrifuged (400 g; 4 °C; 5 min), the pellet was washed with 1 mL of PBS and centrifuged as mentioned earlier. The fluorescence intensity of 10,000 events was analyzed in FL-1 channel on BD FACSCalibur (Becton–Dickinson, USA) flow cytometer. The decrease in fluorescence intensity because of membrane mitochondrial potential loss was analyzed in FL-1 channel and the change of potential membrane (∆ψm) was assessed by comparing fluorescence.

### Reactive oxygen species (ROS) assay

ROS production was monitored by flow cytometry using 2′,7′-dichlorodihydrofluorescin diacetate (DCFH_2_-DA). This dye is a stable non polar compound that readily diffuses into cells and is hydrolyzed by intracellular esterase to yield 2′,7′-dichlorodihydrofluorescin (DCFH_2_), which is trapped within the cells. Hydrogen peroxide or low molecular weight peroxides produced by the cells oxidizes DCFH_2_ to a highly fluorescent compound 2′,7′-dichlorofluorescein (DCF). Thus, the fluorescence intensity was proportional to the amount of hydrogen peroxide produced by the cells. Briefly, HL-60 cells (1 × 10^6^ cells/2 mL/well) were treated with isogarcinol and epigarcinol at different concentrations for 24 h. Thirty minutes before the end of the experiment, the cell culture was treated with DCFH_2_-DA (50 µM) and kept in the dark. Cells were then collected, centrifuged (200 g; 4 °C; 5 min) and the pellet was washed with 1 mL of PBS and centrifuged as mentioned earlier. The pellet was suspended in 500 µL of PBS and the fluorescence was assessed by comparing two fluorescence emission 480 nm/530 nm using a flow-cytometer (BD-LSR).

### Statistical analysis

The viability experiments were done in triplicates and each data point represents the average of at least 3 independent experiments. For other assays, three independent experiments were made and one of them had been chosen as result to post in this study.

## Results

### Cytotoxic effect of *G. ovalifolia* fractions on HL-60 and PC-3 cells

**Fig. 1:**
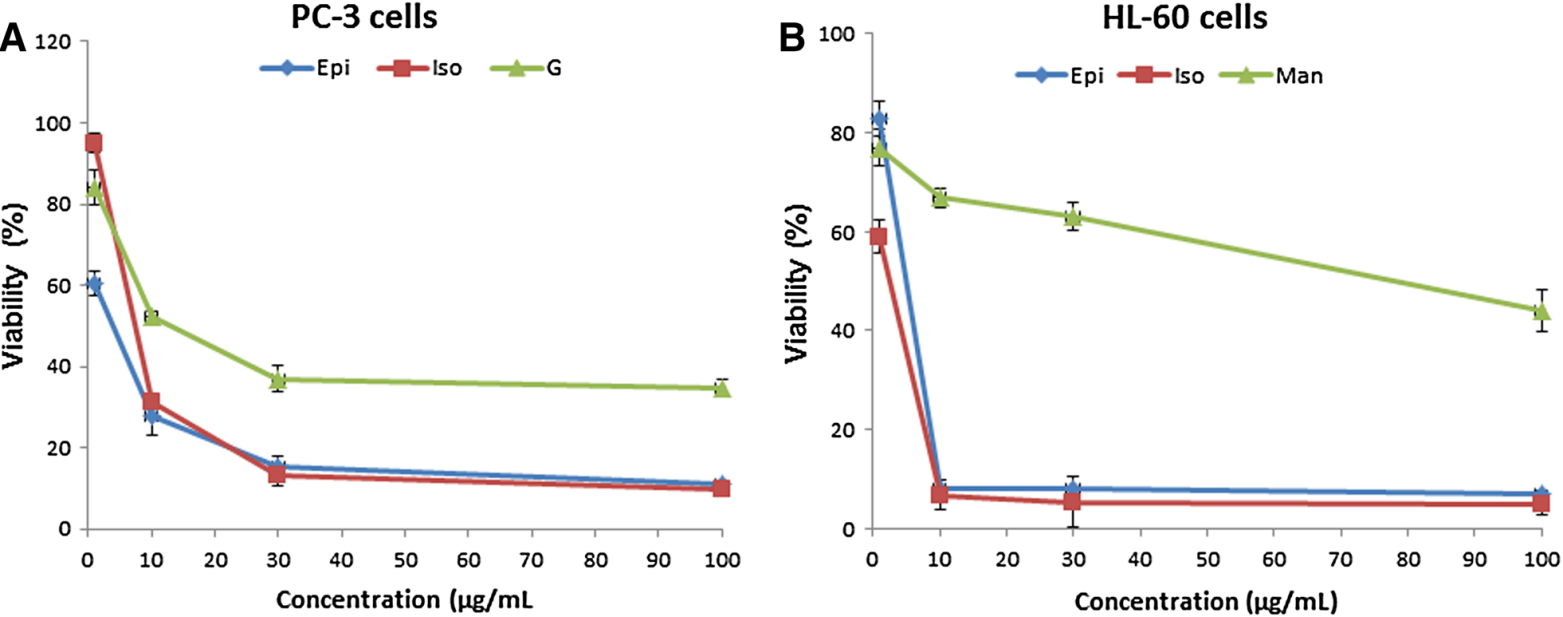
Inhibitory effects and cytotoxicity of *G. ovalifolia* extracts. PC-3 and HL-60 cells were treated with different concentrations of *G. olivalifolia* fractions and extract for 48 h in triplicate. Cell viability was determined by MTT assay and indicated in percentage of cell viability of tree independent assays

To investigate the effect of *G. ovalifolia* fractions and extract on HL-60 and PC-3 cell proliferation, cells were treated with different concentrations for 48 h. The cytotoxic effect of these fractions and extract *G. ovalifolia* on HL-60 and PC-3 cells was evaluated by MTT assay. As shown in Fig. 1, the addition of isogarcinol and epigarcinol to the cultured cells significantly inhibited the proliferation of these cells in a concentration-dependent manner (Fig. 1a, b).

The fifty percent inhibitory concentration (IC_50_) after 48 h were 7, 8 and 12 μg/mL for HL-60 cells respectively for epigarcinol; isogarcinol and unknow molecule while for PC-3 cells these value are 7, 4 and 76 μg/mL respectively for epigarcinol; isogarcinol and manniflavanone (Table 1). These results indicated that epigarcinol; isogarcinol isolated from *G. ovalifolia* effectively inhibited the proliferation of both HL-60 and PC-3 cells and the antiproliferative activity of HL-60 cells greater than that of PC-3 cells. These two molecules (epigarcinol and isogarcinol) were used to continue the study.

**Table 1:** Fifty percent inhibition of molecules isolated from *G. ovalifolia*

Cell	IC_50_ (µg/ml)			
	Epigarcinol	Isogarcinol	Manniflavanone	Unknown
PC-3	7 ± 1.9	8 ± 2.6	>100	12 ± 3.4
HL-60	7 ± 1.3	4 ± 2.1	76 ± 2.3	>100

Results are expressed as mean ± SD; n = 3; Epi:7-epigarcinol; Iso: Isogarcinol; Man: manniflavanone; G: molecule with structure need to be elucidated

### Morphology changes of HL-60 cells

**Fig. 2:**
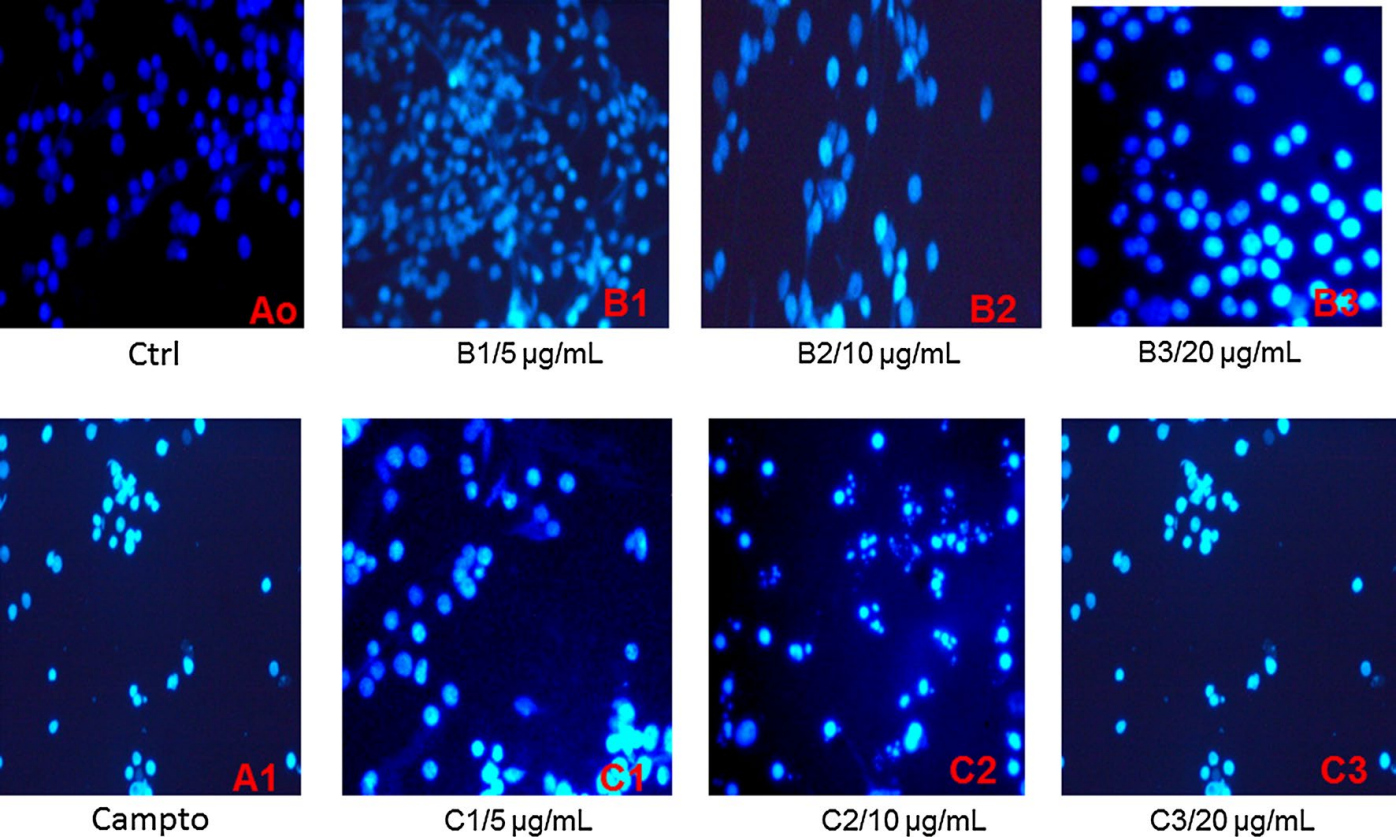
Effect of epi-garcinol and isogarcinol on nuclear morphological changes of HL-60 cells. After 24 h of treatment follow by staining with Hoechst 33258, the cells are incubated for 30 min and observed under fluorescence microscope. Olympus, Tokyo, Japan; magnification ×200). (Ao): Control; (A1): Camptotecin (2 µM); B: Epigarcinol (B1: 5 µg/mL; B2: 10 µg/mL; B3: 20 µg/mL); C Isogarcinol (C1: 5 µg/mL; C2: 10 µg/mL; C3: 20 µg/mL)

To further confirm that cell apoptosis was induced by isogarcinol and epigarcinol from *G. ovalifolia* in HL-60 cells, a morphological examination was performed by Hoechst staining. As shown in Fig. 2, the nuclear Hoechst staining of the control cells was slightly blue and homogeneous, while cells treated with the two molecules exhibited morphological features of apoptotic cells, such as condensed chromatin with bright nuclear Hoechst staining. Under fluorescence microscope, the treated cell displayed an increase of cell damage and the level damage become higher when the concentration of the molecules is raised. Taken together, the obtained results indicate the induction of apoptosis in HL-60 cells after 24 h treatment with isogarcinol and epigarcinol.

### Effect of B and C fractions on intracellular reactive oxygen species in HL-60 cells

**Fig. 3:**
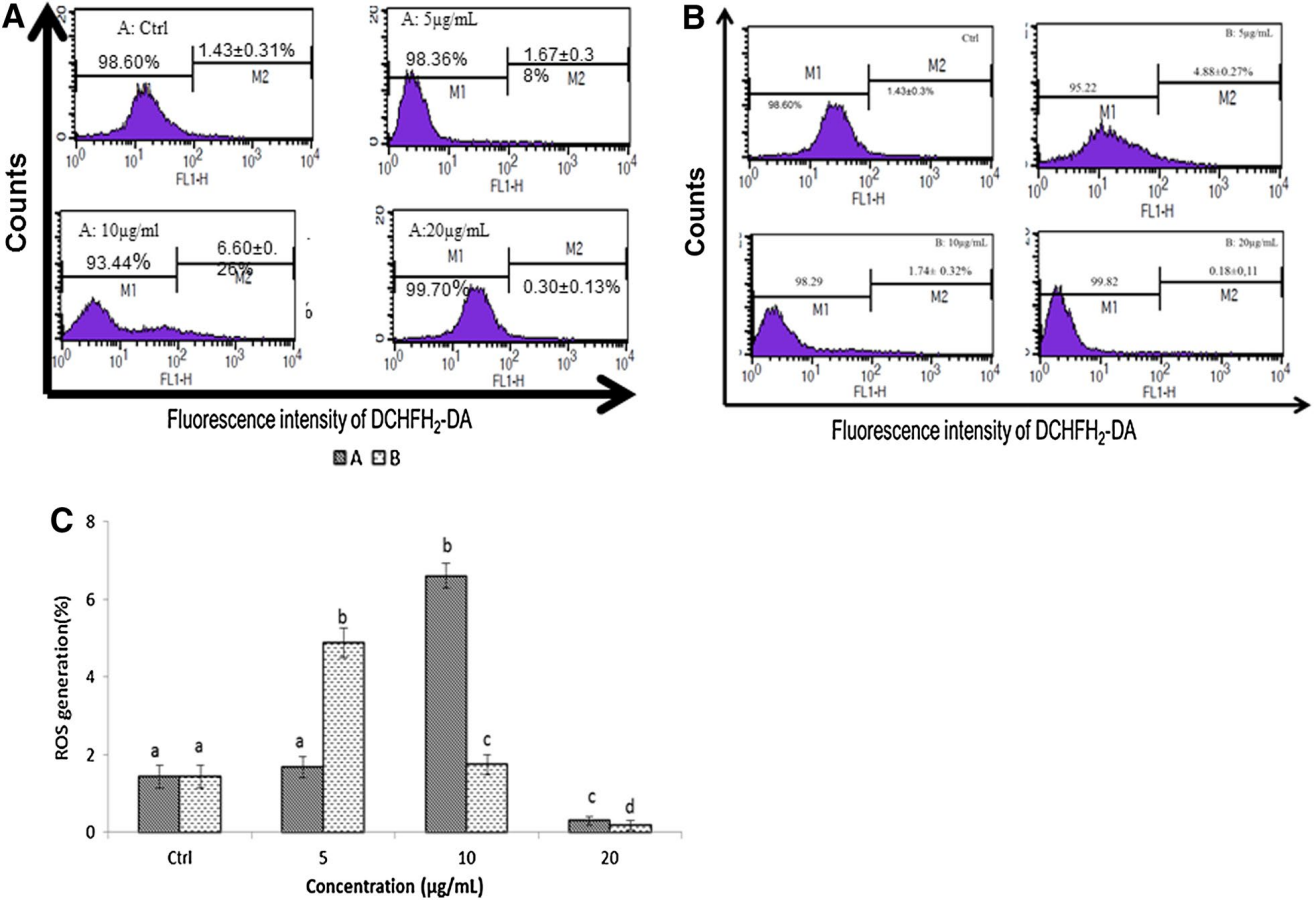
Variation of ROS production of HL60 cells after treatment and analyzed by BD-FACS Caliber flow-cytometer; values are expressed as means ± standard error (n = 3). Ctrl: Control; 5; 10; 20µg/mL. Values affected with different letters are significantly different (p < 0.05) from the control; **A** isopigarcinol; **B** epigarcinol; **C** distribution of ROS fluorescence of isogarcinol and epigarcinol

The generation of intracellular ROS is always associated with MMP disruption and cell apoptosis. To investigate this association, we examined the levels of ROS in HL-60 cells treated with epigarcinol and isogarcinol. ROS was monitored by the oxidation-sensitive fluorescent dye 2′,7′-dichlorofluoresceindiacetate (DCFH_2_-DA). A slight increase in DCF fluorescence was detected in the treated cells with both epigarcinol and isogarcinol (Fig. 3). A slight increase of fluorescence 4–6 times higher than the control was found after treatment of cells with isogarcinol (10 µg/mL; Fig. 3A) and epigarcinol (5 µg/mL; Fig. 3B). At the concentration of 20 μg/mL, both epigarcinol and isogarcinol showed no ROS production this can be explained by the fact that at higher concentration, these two molecules act as antioxidant which neutralized and fixed ROS produced by the cells (Fig. 3C).

### Mitochondrial membrane potential (ΔΨm)

**Fig. 4:**
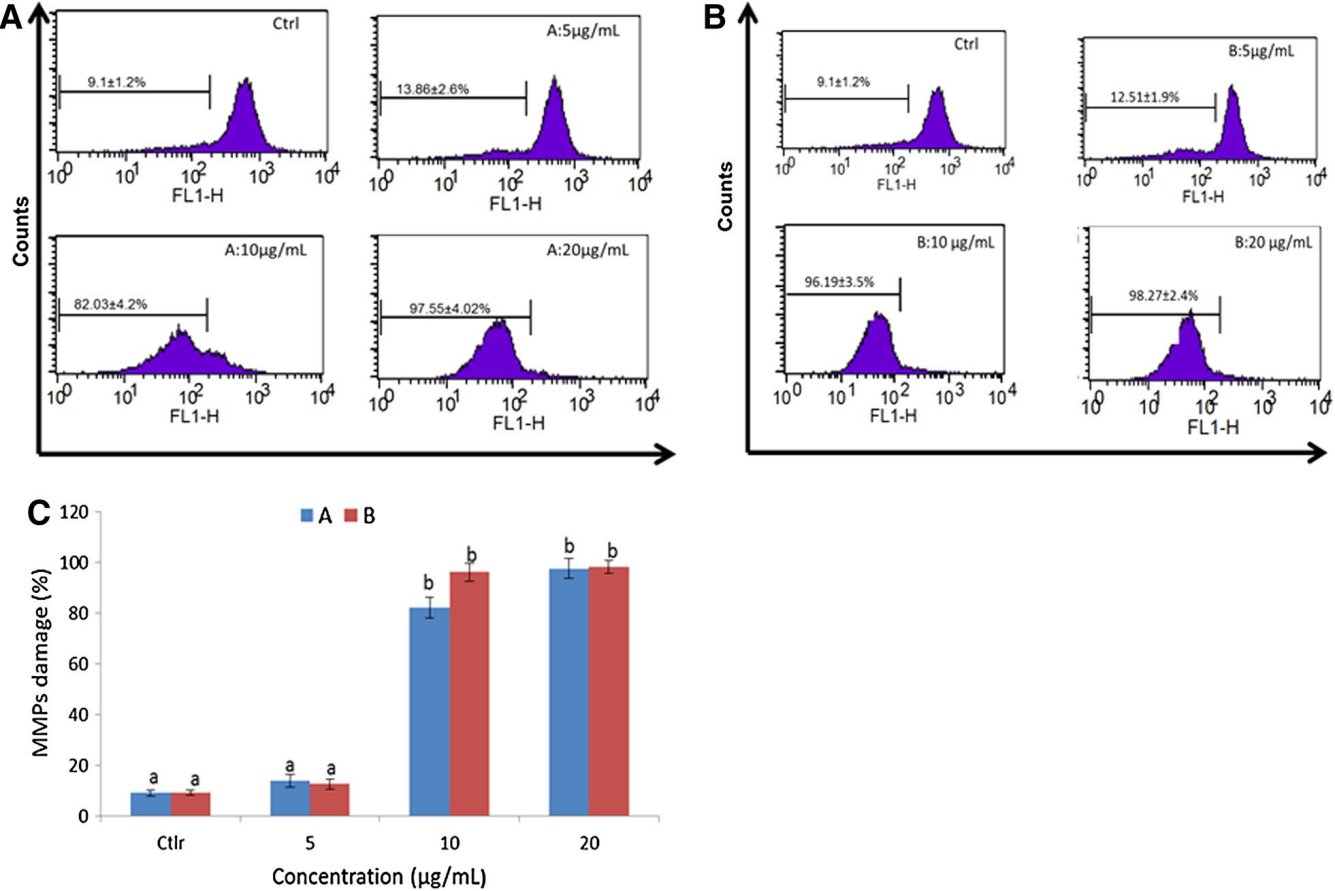
Cell cycle analysis of HL-60 cells after treatment with molecules and analyzed by BD-FACS Caliber flow-cytometer. Ctrl: Control; 5; 10; 20 µg/mL. *Values* are expressed as means ± standard error (n = 3). *Values* affected with different letters are significantly different (p < 0.05) from the control; **A** isogarcinol; **B** epigarcinol; **C** distribution of fluorescence of isogarcinol and epigarcinol

The depletion of ΔΨm is one of the earliest intracellular events that occur following the induction of apoptosis. To determine whether an early loss of ΔΨm occurred during treatment with epigarcinol and isogarcinol in HL-60cells, we performed ΔΨm measurement using Rh-123 staining. In the untreated control cells, almost all cells were functionally active with high Rh-123 fluorescence and the percentage of mitochondrial damage was still low (9.11 %) (Fig. 4). The percentage of mitochondrial potential loss increased from 13.86 to 97.55 % for B fraction and 12.55 to 98.27 % isogarcinol when their concentration raised between 5 and 20 μg/mL (Fig. 4B). At 20 μg/mL, both epigarcinol and isogarcinol isolated from *G. ovalifolia* caused mitochondrial damage and hence the decrease of mitochondrial membrane potential up to 98 % corresponding of 10 fold compare to the control (Fig. 4B). The loss of ΔΨm is largely due to the opening of mitochondrial permeability transition pores (PTP), which conduit the leakage of cytochrome c and pro-apoptotic proteins from mitochondria to the cytosol.

### Effect of fractions of *G. ovalifolia* on cell cycle distribution

**Fig. 5:**
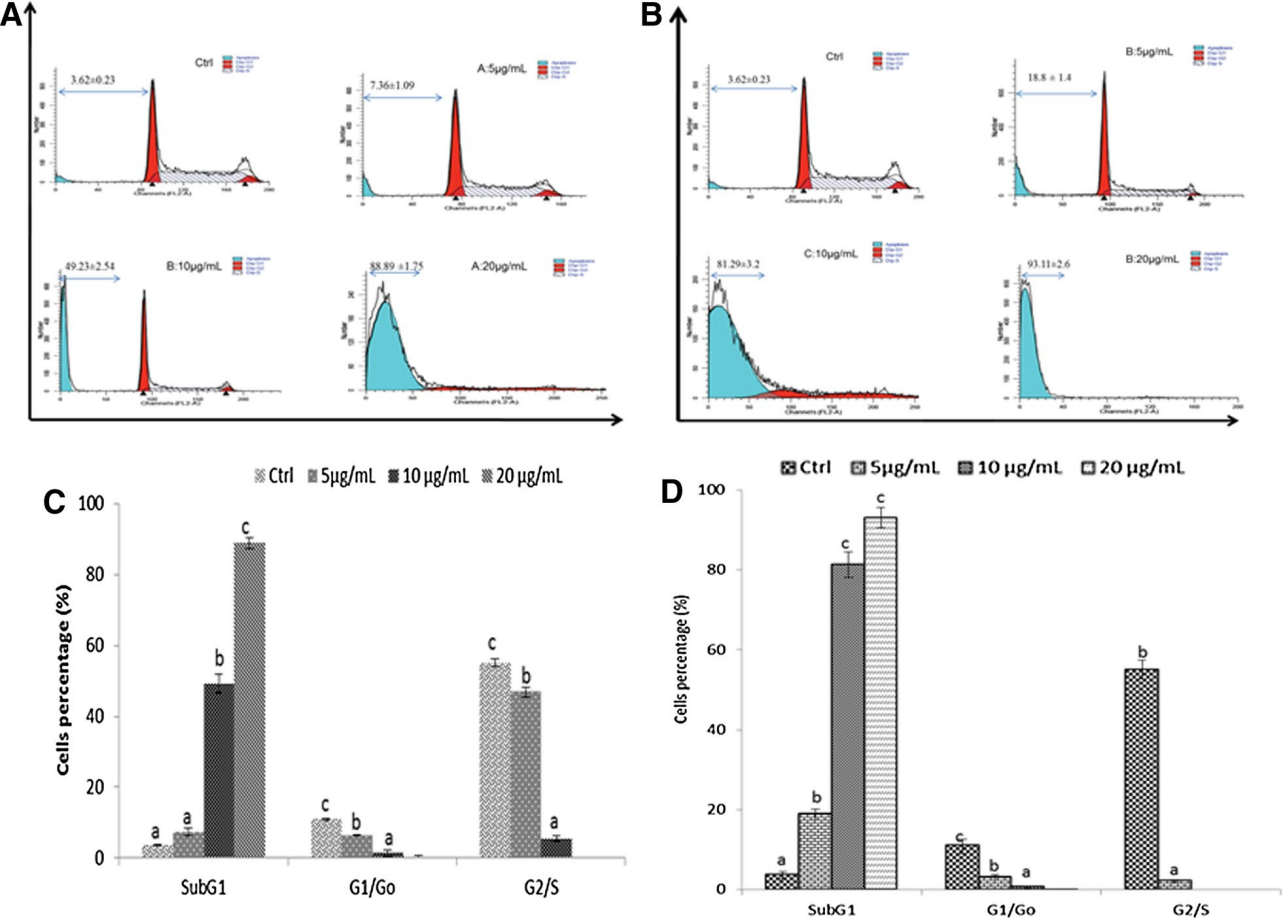
Cell cycle analysis of isogarcinol on HL-60 cells. After 24 h, treated cells were incubated with RNAse (40 µg/mL), stained with propidium iodide (25 μg/mL), and analyzed by BD-FACS Caliber flow-cytometer; Values are expressed as means ± standard error (n = 3); *Values* affected with different letters are significantly different (p < 0.05) from the control; **A** isogarcinol; **B** epigarcinol, **C** cells distribution in different phases of isogarcinol; **D** cells distribution in different phases of Epigarcinol

Quantitative analysis of DNA content with flow cytometry was conducted in order to determine whether the *G. ovalifolia* fractions was associated with the induction of cell cycle arrest and the distribution of cells in different phases of the cell cycle after 24 h exposure. After 24 h treatment with isogarcinol and epigarcinol, the HL-60 cells exhibited concentration dependent increase in hypo diploid sub-G_1_ DNA fraction (<2nDNA). The results revealed an increase in the sub-G1 apoptotic fraction in both molecules from 7.37 % (5 µg/mL) to (88.89 %) 20 µg/mL (epigarcinol) and 18.8 to 93.11 % (isogarcinol) when compared to the control (3.62 %) (Fig. 5). The G1/Go and G_2_/M phases block in HL-60 cells decrease significantly as result of the treatment with *G. ovalifolia* fractions when compared to vehicle control (Fig. 5B). An increase in sub G1 fraction is usually associated with apoptosis as a result of DNA fragmentation [27]. Both molecules isogarcinol and epigarcinol isolated from *G. ovalifolia* showed an apoptosis at 10 µg/mL (49.23 and 81. 29 %, respectively for epigarcinol and isogarcinol compared to the control. In the experiment to measure the change in the cell cycle of HL-60 cells after epigarcinnol and isogarcinol (10 µg/mL), the G_2_/M population was found to be drastically shifted to the sub-G_1_ population, while the significant reduction in the G_o_/G_1_ (Fig. 5C). Moreover, in the flow cytometry study, a sub-G_1_ peak, which was considered as an indicator of cell apoptosis, was clearly observed after the treatment with epigarcinol and isogarcinol at the concentration higher than (5 µg/mL) (Fig. 5A, B). These results suggested that HL-60 cells treated with the two molecules underwent the typical apoptosis. All these indicated that epigarcinol and isogarcinol arrested HL-60 cells at the G_2_/M phase, and primarily stimulated the cells from the G_2_/M phase to the sub-G_1_ phase of apoptosis.

## Discussion

Different types of cancer chemopreventive agents, including natural substances and pharmaceutical compounds, have been investigated for efficacy and efficiency in vitro and in vivo. The induction of apoptosis is known to be an efficient strategy for cancer therapy. Recently, extracts and molecules prepared from a variety of plants were demonstrated to possess the ability in triggering the apoptotic pathway [28]. Our study investigated the in vitro effects of epigarcinol and isogarcinol from *G. ovalifolia* extract on cell growth, morphology, cell cycle of HL-60 cancer cell line and the mechanism of apoptosis induced these two molecules.

As commonly known, naturally polyphenols such as garcinol and isogarcinol exhibited cytotoxic activity against HL-60 and PC-3 cells. The two molecules inhibited the proliferation of these two cells in a dose-dependent manner (Fig. 1) with the IC_50_ varying between 5 and 12 μg/mL (Table 1). Several studies demonstrated the growth inhibition properties of garcinol on different cells cancer including breast, lung, kidney, hepatocellular carcinoma, pancreatic [29–31]. The antiproliferative effects of isogarcinol was shown on HCT116 and CCRF-CEM cell lines [32].

It is also well known that in apoptosis, the earliest recognized morphological changes are compaction and segregation of the nuclear chromatin, with the result of chromatin margination and condensation of the cytoplasm. Our study demonstrated that epigarcinol and isogarcinol induced apoptosis of HL-60 cells as evidenced of alteration of cell morphology, DNA fragmentation, mitochondrial depolarization. After treatment of HL-60 cells with epigarcinol and isogarcinol for 24 h, a representative population of apoptotic cells was also observed mainly at 10 and 20 µg/mL, suggesting a rapid mechanism of apoptose induction that is gradually overwhelmed by the activation of apoptotic pathways. In the present study, the treated cells displayed significant apoptosis-related morphological alterations, such as apoptotic body formation and chromatin condensation. The effects of garcinol in tumor cells have been studied; its mechanism of action is elucidated for several cancer cells lines [29, 33]. The mechanistic cytotoxic activity of garcinol similar to isogarcinol which may include downregulation of MMP-9, IL-8, PGE-2, and VEGF, markers of angiogenesis and metastasis in pancreatic cancer cell lines, Panc 1 and BxPC3 and induction of ROS through death receptor, mitochondrial and modulating GADD153 [34, 35].

ROS are involved in several different cellular processes ranging from apoptosis and necrosis to cell proliferation and carcinogenesis [36, 37], and the mitochondrion is a very important site of ROS production. Mitochondria are a source of ROS during apoptosis and reduced mitochondrial membrane potential leads to increase generation of ROS and apoptosis [38] ROS can induce and maintain the collapse of Δψm, leading to cellular damage through the oxidation of lipids and proteins, resulting in apoptotic cell death [39]. Controversially, other studies estimated that both mitochondria and ROS production seem to be involved in necrotic cell death [37, 40]. Whether the treated cells undergo apoptosis or necrosis depend on the concentration of ROS and the time of exposure to ROS [41]. Our results showed that epigarcinol and isogarcinol inhibited growth of HL-60 cells and induced apoptosis and ROS generation not in a dose-dependent manner. Because cell cycle and apoptosis are involved in the regulation of cell growth, they were detected with a flow cytometer 24 h after the treatment with epigarcinol and isogarcinol in our study. The results showed that after treated with the two molecules for 24 h, the cells arrested at G_o_/G_1_ and G_2_/M phases decreased respectively and the increase of sub-G_1_, suggesting that these molecules induce significant apoptosis.

Even though apoptosis involves both intrinsic and extrinsic pathways, tumors arise more frequently through the intrinsic pathway than the extrinsic pathway because of its sensitivity [42]. The main intrinsic pathway is characterized by mitochondrial dysfunction, with the release of cytochrome c. Since mitochondria have the ability to activate the cellular apoptotic program directly, they are considered the major cellular component in the intrinsic way of apoptosis [43]. The mitochondria play an important role in the progression of apoptosis. In the study, the decrease of Δψm was enhanced by increasing concentration of epigarcinol and isogarcinol. These results suggest that the two molecules indeed induced apoptosis in HL-60 cells A decrease in Δψm disrupts the outer mitochondrial membrane and causes the opening of the permeability transition pore, followed by the lease of pro-apoptotic factors, and resulting in subsequent apoptosis [44–47]. Other studies demonstrated the inhibition of CBP/p300 acetyltransferase activity by garcinol and several multiple biological effects in cancer cells, including the activation of DNA damage signaling and the induction of chromatin regulators such as TIP60 and SUV420 [47].

## Conclusion

The bioassay-guided approach to the processing of *G. ovalifolia* led to the isolation and purification of epigarcinol and isogarcinol. We demonstrated that epigarcinol and isogarcinol from *G. ovalifolia* can inhibit the proliferation of HL-60 cells and this inhibitory effect was related to G2/M phase arrest and apoptosis the mitochondrial pathway. The cytotoxic results obtained from our work represent new insight into the traditional use of *G. ovalifolia* as an anticancer plant. Epigarcinol and isogarcinol may thus warrant further in vivo studies so as to develop it as a successful natural compound that could be used for the treatment in leukemia after determine the entire mechanism of its cytotoxicity.
